# Tryptophan–Serotonin–Melatonin Pathway and It Contribution to Mood and Cognitive Changes During Sleep Deprivation?

**DOI:** 10.1192/j.eurpsy.2026.11986

**Published:** 2026-07-31

**Authors:** M. Sochal, A. Wojtera, M. Ditmer, S. Turkiewicz, F. F. Karuga, P. Białasiewicz, D. Strzelecki, A. Gabryelska

**Affiliations:** 1 Department of Sleep Medicine and Metabolic Disorders; 2 Department of Affective and Psychotic Disorders, Medical University of Lodz, Lodz, Poland

## Abstract

**Introduction:**

Sleep deprivation (DS) is a sleep reduction, arising from voluntary or external factors. While acute DS can transiently alleviate depressive symptoms, it negatively affects cognitive functions. The underlying neurochemical mechanisms are not fully understood, but may involve the serotonergic and melatoninergic pathways, sharing the precursor tryptophan (TP).

**Objectives:**

The study aimed to evaluate the effects of a single night of DS on the peripheral concentrations of serotonin (5-HT), TP and melatonin (MLT), and their associations with cognitive performance and mood.

**Methods:**

Polysomnography (PSG) and actigraphy-confirmed DS were performed in 80 healthy participants. Blood samples, Montgomery–Åsberg Depression Rating Scale (MADRS), and Bimanual Eye-Hand Coordination Test (BEHCT) were collected before and after PSG or DS. Peripheral levels of TP, 5-HT, and MLT were measured via ELISA. Responders (RE) were defined as individuals with post-DS MADRS<8 or with overnight mood improvement; others were classified as Non-responders (NR). Approved by the local bioethics committee (RNN/302/20/KE). Funded by the Ministry of Education and Science (Poland) SKN/SP/629918/2025.

**Results:**

In the whole sample, TP (44.9 [30.4–68.2] vs. 30.8 [19.9–56.9] μmol/L; p=0.024) and MLT (457.0 [391.7–511.2] vs. 423.6 [392.7–464.2] pg/mL; p=0.043) significantly decreased post-DS, while 5-HT did not change significantly (p=0.120). Among NR, 5-HT increased (63.8 [30.7–86.7] vs. 76.6 [53.8–120.3] ng/mL; p=0.043) and MLT decreased (459.3 ± 73.2 vs. 423.6 ± 57.1 pg/mL; p=0.024); TP showed a nonsignificant downward trend (p=0.055). No significant changes were found in RE. A positive correlation was observed between 5-HT and MLT and BEHCT errors in the full group (R=0.25; p=0.028 and R=0.28; p=0.018). In RE, TP negatively correlated with BEHCT errors (R=–0.32; p=0.029), while MLT showed a positive correlation (R=0.41; p=0.005). No associations were observed in NR.

**Conclusions:**

One night of DS reduces circulating TP and MLT, disrupting the circadian rhythm, with no overall change in 5-HT. Only in NR, 5-HT increased while MLT decreased significantly. The results imply that mood improvement after DS is not directly tied to TP–5-HT–MLT alterations, suggesting involvement of alternate pathways. Conversely, cognitive deficits post-DS associate with elevated MLT and 5-HT, and may reflect neurochemical dysregulation affecting vigilance.

**Disclosure of Interest:**

None Declared

